# Impact of COVID-19 Vaccination on Women During Pregnancy and Breastfeeding

**DOI:** 10.7759/cureus.38547

**Published:** 2023-05-04

**Authors:** Usha Kumari, Raj Kishor Sharma, Archana Sinha, Minakshi Sinha, J R Keshari

**Affiliations:** 1 Biochemistry, Indira Gandhi Institute of Medical Sciences, Patna, IND; 2 Microbiology, Patna Medical College, Patna, IND; 3 Obstetrics and Gynaecology, Indira Gandhi Institute of Medical Sciences, Patna, IND; 4 Gynecology, Indira Gandhi Institute of Medical Sciences, Patna, IND

**Keywords:** anti-sars-cov-2 vaccine, neonatal health, pregnancy outcome, breastfeeding, covid-19 vaccination

## Abstract

Rapid development of anti-SARS-CoV-2 vaccinations in the late 2020s has significantly altered the trajectory in which the virus affects various patient demographics, especially the most susceptible ones. In light of ethical and conceptual safety considerations, pregnant women were initially barred from participating in clinical studies for the coronavirus disease 2019 (COVID-19) vaccination programs. However, the steady accumulation of reliable observational data from cohorts of pregnant women who received vaccinations enabled the research establishments to quickly address a number of open questions. Still, more than a year after vaccines were widely available, the safety concerns of expectant or nursing mothers are cited as the primary justification for refusing COVID-19 vaccination, and notably, the rate of vaccination in the said populations is known to be consistently lower than those of the general populace.

In light of such a scenario, we have made an attempt to garner relevant studies that evaluated the effect of COVID-19 vaccination on pregnant and lactating mothers which may prove to be supporting evidence for its wide usage among the said population.

## Introduction and background

The rapid development and administration of anti-SARS-CoV-2 vaccines in the late 2020s have significantly altered how the virus affects different patient segments, particularly the most vulnerable [1]. Due to ethical and hypothetical safety issues, pregnant women were previously disallowed from participating in clinical studies for the coronavirus disease 2019 (COVID-19) vaccination [2]. Yet, steady accumulation of reliable empirical evidence from cohorts of pregnant women who received vaccinations enabled the research establishment to quickly address a number of open questions. However, more than a year after vaccines were made widely available, safety concerns of expectant or nursing mothers are still cited as the primary justification for refusing COVID-19 vaccination, and their vaccination percentages are markedly lower than those of the general public of a comparable age group [3,4] This viewpoint has been helped, in part, by the notion that suggestions for the use of anti-SARS-CoV-2 vaccines in pregnant and nursing women have been periodically revised and updated by various national and international regulatory agencies [5]. The early and appropriate caution was swiftly replaced by advice to vaccinate both pregnant and breastfeeding women that were first more lenient and eventually encouraging.

Pregnancy-related physiological, mechanical, and immunologic shifts may affect a woman's risk of contracting severe acute respiratory syndrome coronavirus 2 (SARS-CoV-2) [6]. The primary symptoms of the said disease are caused by reduced microcirculatory functioning; hence, pregnant women who have the infection are more likely to experience preeclampsia-like symptoms, require hospitalization, and may also require admittance to an intensive care unit. Infants born to infected women with a more serious clinical presentation may have a worse prognosis, primarily due to neonatal morbidity and mortality linked to prematurity, as the prevalence of obstetrical problems, such as preterm birth, which appears to be connected to the intensity of the infection [7]. Therefore, it would seem appropriate to vaccinate expectant women against SARS-CoV-2 [8].

Due to the exclusion of pregnant women from clinical trials analyzing vaccines, there is currently no complete indication regarding the efficacy and safety of COVID-19 vaccines. However, studies conducted to date have enabled the detection of a significantly lower risk of developing SARS-CoV-2 infection among vaccinated pregnant females than unvaccinated counterparts. At a global level, many tens of thousands of pregnant females have received the COVID-19 vaccine to date, with no indications of adverse events above that for the general population [9]. The effectiveness of vaccination during nursing is likewise thought to be comparable to that in women who are not pregnant. There is currently broad agreement that there is no scientific evidence to show potential damage to infants breastfed by vaccinated women [10].

In such a context, an attempt was undertaken to present literature focusing on various studies that evaluated the effect of COVID-19 vaccination on pregnant and lactating mothers which may prove to be supporting evidence for its wide usage among the said population.

## Review

Current status of COVID-19 vaccination in pregnant and lactating women in India and the World

In India, at present three vaccines have received approval for restricted use in emergency situations. One of them is an inactivated vaccine (Covaxin) and the other two are based on nonreplicating viral vector platforms (Covishield and Sputnik V) [11]. If a pregnant woman chooses vaccination, she may receive the shot at any point in her pregnancy. The risks of COVID-19 infection during pregnancy, the advantages of vaccination, and the possible adverse effects of immunization should all be explained to pregnant women to help them make an informed decision about becoming immunized.

The Ministry of Health and Family Welfare (MoHFW), Government of India on July 2, 2021, provided approval for the vaccination of pregnant women against COVID-19 [12]

In India, the decision to opt-in for vaccination is left to the pregnant woman after informing her about the pros and cons of vaccination [13].

The acceptability of the COVID-19 vaccination among expectant moms and mothers of young children varied significantly by geographic region, according to the reported research [14]. In India, the Philippines, and Latin America, acceptance was noted to be higher than 60% for pregnant women, higher than 78% for non-pregnant women for themselves, and higher than 75% for mothers for their offspring. In contrast, women in the US and Russia continually showed lower levels of acceptability, optimism in the effectiveness and safety of the COVID-19 vaccine, anticipated relevance of receiving the vaccine, and public confidence.

Approximately 71.5 % of participants in a worldwide study performed in the year 2020 in 19 countries reported that they were likely to obtain the COVID-19 vaccine, while acceptance levels varied from 90% in China to lower than 55% in Russia. It was also noted that middle-income nations like Brazil, India, and South Africa had a reasonably high possibility of acceptance [15]. The higher acceptability among middle-income nations raises the possibility that the past burdens of other infectious diseases have contributed to both the increased awareness of COVID-19 risk and the increasingly positive mindset toward vaccination.

Factors affecting COVID vaccination/factors causing refusal of vaccination

The choices of pregnant women to get the COVID-19 vaccine can be negatively impacted by ambiguity over the vaccine's consequences on pregnancy, which can cause hesitation about vaccination [14]. Hesitancy regarding vaccination is defined as a delay in acceptance or refusal of vaccination despite the availability of vaccine services [16]. A number of varying personal or contextual considerations, such as social context, vaccine accessibility, satisfaction following vaccination, apparent vaccine safety, the relative advantage of vaccination, religious belief systems, ignorance, and demeanor toward vaccination, have an impact on the multidimensional construct of vaccination hesitancy [17-19]. Age, gender, educational qualification, health care insurance coverage, level of faith in government information, perceived vulnerability to COVID-19, anticipated benefits and disadvantages of vaccination, and mindset toward vaccine are some of the socio-demographic factors that also have an impact on vaccination hesitancy [20].

According to the survey responses collected from 16 nations, the overall vaccine acceptance rate was 52.0% in pregnant women while it was 73.4% in the non-pregnant women. Vaccine acceptance was the highest in India, the Philippines, and all countries enrolled from Latin America. However, it was lowest in Russia, the United States, and Australia [14]. Another study on pregnant and nursing women in six European nations found that 40-50% of them were reluctant to get the COVID-19 vaccination (HACV) [21]. A study in 11 countries in Asia, Africa, and South America found that poor opinions of vaccination advantages and perceptions that new vaccinations are riskier were connected with the female population, Muslim community, having a non-healthcare-related work, and not having a flu vaccination over last 12 months [22]. Political association and the perceived risk of contracting COVID-19 in the upcoming year were also used to predict vaccine reluctance [23]. 

Anticipated illness in expecting mothers with SARS-CoV-2 infection

Pregnant women are considered to be more prone to acquiring severe illness and death due to COVID-19 in comparison to nonpregnant women of a similar reproductive age group; they remain at higher risk of adverse pregnancy outcomes, such as preterm birth [24,25].

It is imperative to get comprehensive knowledge on how COVID-19 affects pregnancy-related outcomes as compared to results in uninfected pregnant women. In this quest, a study was conducted to assess the risks of COVID-19 infection during pregnancy on maternal and newborn outcomes in comparison to concomitantly pregnant people who were not infected [25]. A total of 1424 pregnant women (all with a mean age of 30.2 ±6.1 years and 706 pregnant women (all with a diagnosis of COVID-19) were included in the study. Among the enrolled participants, 48.6% with a COVID-19 diagnosis and 40.2% without were overweight in the early stages of pregnancy. Preeclampsia/eclampsia, serious infections, intensive care unit admission, maternal mortality, preterm delivery, medically recommended preterm birth, and severe neonatal morbidity index along with severe perinatal morbidity and mortality index were all more common in the enrolled females with a COVID-19 diagnosis. A higher risk of severe maternal complications and newborn difficulties was linked to fever and shortness of breath for any length of time. Only maternal morbidity and preeclampsia were maintained as a greater risk for asymptomatic women with a COVID-19 diagnosis. Thirteen percent of the neonates of the women who tested positive also were noted to be COVID-positive. Elevated risk for newborn testing positive was linked to cesarean mode of delivery but not breastfeeding. Breastfeeding was not linked to an increase in the rate of test-positive newborns because SARS-CoV-2 has not been detected in breast milk [26]. The findings of the study indicated that when pregnant women with and without a diagnosis of COVID-19 were evaluated, severe maternal morbidity and mortality as well as neonatal problems were consistently and significantly increased. The findings would warn expectant women and healthcare professionals to properly follow all advised COVID-19 preventative measures.

Preterm delivery was the most frequently reported unfavorable outcome. According to publications using data from a multitude of settings and designs, a growing prevalence of lower birth weight and cesarean-section (C-section) deliveries was also noted [27,28]. Premature membrane breach, perinatal mortality, stillbirth, miscarriage, hypertension, fetal growth limitation, coagulopathy, and other obstetric problems and consequences were uncommon but noticeable [29]. However, a study from London indicated that stillbirths may rise as a direct or indirect result of the pandemic, but observational data did not demonstrate that COVID-19 directly elevated the risk of these events [30].

Pregnant women with severe COVID-19 may advance a preeclampsia-like syndrome without anomalous ratios of soluble fms-like tyrosine kinase 1 to placental growth factor (sFlt-1/PIGF) and uterine artery pulsatile index (UtAPI) scores classical of normal preeclampsia, according to a prospective cohort study [31]. Although the virus was shown to infect the placenta, most individuals were asymptomatic or very slightly symptomatic [32,33].

Maternal mortality/morbidity among non-vaccinated pregnant women

A study compared the severity of symptoms and results among COVID-19 participants who were and were not vaccinated [34]. The effectiveness was higher among vaccinated patients than it was among the unvaccinated ones. In contrast to patients who had received vaccinations, non-vaccinated patients had considerably greater rates of unfavorable outcomes like hospitalization, ICU admission, and mortality. It was revealed that COVID-19 vaccination was successful and beneficial for lowering the severity of the condition. Similar results were reported elsewhere wherein recovery among the vaccinated mass was greater (80%) than in non-vaccinated patients (38.6%) [35]. In contrast to vaccinated patients, who experienced hospitalization ICU admission and mortality was found in a smaller number of cases, the recurrence of poor consequences in non-vaccinated patients was significantly higher.

PregCovid is a research study on pregnant and postpartum women with SARS-CoV-2 infection conducted by the Government of India [36]. The program's objectives were to examine the socio-demographics, clinical manifestations, and reproductive traits of women who were pregnant or recently gave birth and had been diagnosed with SARS-CoV-2 infection, to ascertain the fetal and pregnancy results in women who have SARS-CoV-2 infection and investigate the impact of treatment, to determine the percentage of SARS-CoV-2 infection in pregnancy that is transmitted from the mother to the fetus. A total number of 6625 cases were enrolled in the registry [36].

Morbidity and mortality among neonates born to COVID-infected mothers

In comparison to the national average of 10.2%, 12.9% of the 3,912 children with known gestational ages born to females who had SARS-CoV-2 infection were preterm (37 weeks). Approximately 2.6% of the 610 newborns having laboratory results had positive SARS-CoV-2 outcomes, mostly those born to pregnant women who were already infected [37]. This result covers consequences for females hospitalized as well as those not hospitalized at the time of infection, which is in accordance with previous CDC publications indicating greater rates of preterm births among females hospitalized during the time of SARS-CoV-2 infection [38]. A comprehensive, systematic review of SARS-CoV-2 infection in pregnancy also revealed a rise in premature deliveries [39]. A longitudinal prospective cohort study enrolling 253 infants, however, discovered no disparity in the percentage of preterm births or infant ICU admissions between those born to females with positive SARS-CoV-2 test outcomes and those born to females with presumed SARS-CoV-2 but negative test results [40]. The discrepancy in the outcomes between these two different studies may be due to distinctions in case assessment, research methods, data analysis, and study population. To determine the true risk of preterm birth, researchers comparing pregnant females with and without COVID-19 are necessary.

Impact of non-vaccination of pregnant women on stillbirth /neonatal health

Even though stillbirth was a relatively uncommon event altogether, a COVID-19 diagnosis recorded during the hospitalization for delivery was linked to a higher chance of stillbirth in the United States, with the link being greater during the time when the Delta variant predominated. Inflammation and placental hypoperfusion were reported to be prevalent with maternal COVID-19 infection, according to findings from a prior investigation of pregnancies affected by SARS-CoV-2 infection [41]. These observations may help to elucidate the link between COVID-19 and stillbirth. Among births with COVID-19 reported at the hospital delivery, stillbirth was linked to specific underlying illnesses and indicators of maternal morbidity, including the requirement for intensive care. However, it is necessary to conduct more research to determine how COVID-19-related maternal problems affect the risk of stillbirth.

The total stillbirth rate in an investigation (0.64%) for women without COVID-19 during delivery was comparable to the previously reported pre-pandemic stillbirth rate (0.59%) [42]. However, stillbirths occurred in 0.98% of COVID-19-affected deliveries prior to Delta and 2.70% during Delta. There is growing evidence that COVID-19 during pregnancy and stillbirth are related. Despite finding a link between COVID-19 during pregnancy and its association with stillbirth, two meta-analyses failed to account for confounding factors [43]. When variables were taken into account in a prior analysis, which compared females with and without COVID-19 reported at the delivery hospitalization, the probability of stillbirth was not significantly higher [44].

In July 2021, Delta started to predominate among SARS-CoV-2 strains in the US. Compared to earlier versions, the Delta variant was noted to be more contagious and is linked to a higher risk of hospitalization [45]; nevertheless, non-pregnant patients are not more prone to experience poor outcomes when hospitalized [45,46]. In a research study, the phase of Delta preponderance was when the correlation between COVID-19 and stillbirth was strongest. However, there is a need for more research on how SARS-CoV-2 infection, including with the Delta form, affects fetal health to support the said statement. This research underlines the fact that maternal morbidity has an impact on the risk for stillbirth related to COVID-19 and emphasizes the expanding body of data supporting this association. It also shows that the risk has climbed up during the Delta period. It is necessary to conduct additional research from prospective cohort studies to affirm these conclusions, pinpoint the biological basis for the observed elevated risk of stillbirth associated with maternal COVID-19, and compare risks, severity, and presence of maternal risk factors. Further research into the usefulness of vaccines during pregnancy, particularly the prevention of stillbirth, is also necessary. The significance of COVID-19 preventive methods, such as vaccination before or during pregnancy, is most strongly supported by these findings.

Teratogenic potential of COVID-19 vaccines

The Pfizer, Moderna, and Janssen vaccines have not been linked to any teratogenic or fetotoxic side effects of COVID-19 vaccines in animal trials [47,48]. Neonates with structural malformations are born in 3% to 5% of births in the United States, and they are associated with higher infant morbidity and mortality, as well as billions of dollars in costs. The findings of Ruderman et al. indicated that COVID-19 vaccination during early pregnancy is not related to an increased risk of fetal structural defects seen by ultrasound. The existence of a congenital defect detected on ultrasonography was not linked with vaccination during the teratogenic window after adjusting for potential confounders such as age at delivery, nulliparity, chronic hypertension, and hemoglobin A1c level during the first trimester, as inferred by one study [49].

The conclusions obtained in these studies are constrained by the data's retrospective, single-center origin, and the limitations of electronic medical records. Furthermore, because ultrasonography markers are surrogate outcomes and many pregnancies in the data set are ongoing, neonatal outcomes were not routinely available. Given the critical need for safety data on COVID-19 vaccines, these preliminary findings may be informative when contemplating vaccination during early pregnancy. However, the lack of long-term prospective studies warrants comprehensive, multi-centric, and long-term studies to evaluate the teratogenic effects of COVID-19 vaccination. 

COVID-19 and lactating mothers/breast milk as a potential route of transmission of COVID-19

Uncertainty surrounds the potential transmission of new coronaviruses through breast milk. With the exception of Zhu et al. and Wu et al. who detected one positive sample among five tested samples collated from five females and among three samples from three females respectively [50,51], the bulk of milk samples taken from 37 women tested negative for SARS-CoV-2 [52-54]. These case reports and case series involving postpartum women who screened positive for COVID-19 during pregnancy made up the scientific evidence on the prevalence of SARS-CoV-2 in breast milk [50,51]. Breast milk samples taken from a female who had tested positive for COVID-19 on a throat swab were found to contain IgG and IgA SARS-CoV-2 antibodies, according to a reported study [55]. This revealed that breast milk might safeguard against COVID-19 infection, albeit additional proof is required for validation. 

A cross-sectional survey was conducted which included 4455 breastfeeding women who had received a vaccination. Considering the short-term health risks such as fever, exhaustion, or headache which are noted to subside after 72 hours of immunization, their data indicate that vaccination appeared to have little adverse effects on lactation [56,57]. IgG was already identified just one week following the second dosage of the vaccine, while milk IgA levels increased two weeks following the first dose and were evident in 86% of cases in a time span of two weeks after the second dose [58]. Findings from 23 trials, which included 117,552 pregnant women who had received the COVID-19 vaccine, showed that the vaccinated population had a considerably decreased stillbirth rate [59]. A heightened incidence of poor outcomes, such as miscarriage, early gestation at birth, placental abruption, pulmonary embolism, postpartum hemorrhage, maternal mortality, ICU admission, poorer birthweight, or neonatal ICU admission rate, was also not shown to occur.

Public health and medical groups issued recommendations for breastfeeding females who had been diagnosed with SARS-CoV-2 illness, balancing the danger of infection with the well-established advantages of breastfeeding and immediate bonding. Continuing breastfeeding, skin-to-skin touch, and kangaroo care using infection control procedures were all advised by the WHO and UNICEF. Mothers with anticipated or diagnosed COVID-19 should be encouraged to begin or maintain breastfeeding, according to the WHO [60].

## Conclusions

It is safe to administer COVID-19 vaccinations to expectant or nursing mothers owing to their role in minimizing the negative effects of SARS-CoV-2 infection in mothers and their toddlers. Vaccination is temporally contraindicated in situations in which recovery from infection with COVID-19 is postponed for 4 to 8 weeks or 12 weeks, depending on the case, current infection with COVID-19, or when convalescent plasma or anti-COVID-19 monoclonal antibodies are used to treat COVID-19 infection. The advantages of receiving the COVID-19 vaccine during pregnancy surpass the risks for both mothers and newborns. In order to attain the best feasible immunization compliance in this high-risk population group, national and supranational organizations should make every endeavor to deliver explicit and standardized recommendations to expectant and nursing mothers. The vaccination of pregnant women against COVID-19 will be a boon for India and will help in achieving the target of the Sustainable Development Goals, which advocates reducing the global MMR to <70 per 100 000 live births.
